# “That’s just Future Medicine” - a qualitative study on users’ experiences of symptom checker apps

**DOI:** 10.1186/s12910-024-01011-5

**Published:** 2024-02-16

**Authors:** Regina Müller, Malte Klemmt, Roland Koch, Hans-Jörg Ehni, Tanja Henking, Elisabeth Langmann, Urban Wiesing, Robert Ranisch

**Affiliations:** 1 Institute of Philosophy, University Bremen, Bremen, Germany; 2 Institute of General Practice and Palliative Care, Hannover Medical School, Hannover, Germany; 3 Institute of General Practice and Interprofessional Care, University Hospital Tübingen, Tübingen, Germany; 4 Institute of Ethics and History of Medicine, University Tübingen, Tübingen, Germany; 5 Institute of Applied Social Science, University of Applied Science Würzburg- Schweinfurt, Würzburg, Germany; 6 Faculty of Health Science Brandenburg, University of Potsdam, Potsdam, Germany

**Keywords:** Symptom checker, Health apps, mHealth, Digital health, Medical ethics, Bioethics, Interview study, (Self-)Triage

## Abstract

**Background:**

Symptom checker apps (SCAs) are mobile or online applications for lay people that usually have two main functions: symptom analysis and recommendations. SCAs ask users questions about their symptoms via a chatbot, give a list with possible causes, and provide a recommendation, such as seeing a physician. However, it is unclear whether the actual performance of a SCA corresponds to the users’ experiences. This qualitative study investigates the subjective perspectives of SCA users to close the empirical gap identified in the literature and answers the following main research question: How do individuals (healthy users and patients) experience the usage of SCA, including their attitudes, expectations, motivations, and concerns regarding their SCA use?

**Methods:**

A qualitative interview study was chosen to clarify the relatively unknown experience of SCA use. Semi-structured qualitative interviews with SCA users were carried out by two researchers in tandem via video call. Qualitative content analysis was selected as methodology for the data analysis.

**Results:**

Fifteen interviews with SCA users were conducted and seven main categories identified: (1) Attitudes towards findings and recommendations, (2) Communication, (3) Contact with physicians, (4) Expectations (prior to use), (5) Motivations, (6) Risks, and (7) SCA-use for others.

**Conclusions:**

The aspects identified in the analysis emphasise the specific perspective of SCA users and, at the same time, the immense scope of different experiences. Moreover, the study reveals ethical issues, such as relational aspects, that are often overlooked in debates on mHealth. Both empirical and ethical research is more needed, as the awareness of the subjective experience of those affected is an essential component in the responsible development and implementation of health apps such as SCA.

**Trial registration:**

German Clinical Trials Register (DRKS): DRKS00022465. 07/08/2020.

**Supplementary Information:**

The online version contains supplementary material available at 10.1186/s12910-024-01011-5.

## Background

Imagine waking up with a sore throat and other symptoms of a cold. You pick up your smartphone and open the Symptom Checker App (SCA) that a friend just recommended. The chatbot asks you some questions about your symptoms, including duration, intensity of your pain, and other relevant factors. As you lie in your bed, you click through the questions, and after a few minutes, the SCA provides a list of possible causes and a recommendation to stay home for now. While many people have likely had similar experiences in using such apps, little is currently known about subjective experiences of SCA users, including their expectations, motivations or concerns.

SCAs are mobile or online applications that allow lay people to identify possible causes of their symptoms (symptom analysis) and provide recommendations on whether to seek health care (self-triage). Most SCAs are freely available. Typically, a SCA prompts the user to answer a series of questions about their symptoms through a structured questionnaire or chatbot interface. ‘Chatbots are systems that are capable of conversing with users in natural language in a way that simulates the interaction with a real human’ [1]. In the case of SCA, the app asks questions via text that the user is supposed to answer either by selecting predefined choices or in a free-form manner. To be precise, we cannot say that SCAs are like ‘conversational agents’, but rather a kind of questioning tool. When the information is collected, the app generates a list with possible causes along with recommendations, such as seeing a physician in a timely manner or staying at home. The introductory example illustrates that lay people could easily use SCAs to assess their symptoms at home, without having to consult medical professionals. SCAs are part of the increasing global trend of online health information-seeking behavior [2]. The search for information on health and illness outside the traditional health care system that has emerged with the internet is critically examined, for example as an individual ‘lifestyle’ issue [3], its’ influence on the patient-physician relationship [4], or regarding the underlying models of information seeking behaviour [5]. Despite the vast amount of literature on mobile or online health applications in general, there is a lack of (empirical) research focused specifically on SCAs [6].

There are few empirical studies specifically on SCAs, in particular on their diagnostic and triage accuracy. A 2015 study showed that symptom checkers had deficits in symptom assessment and triage advice [7]. Seven years later another study that refers back to Semingran et al. showed that the triage performance of SCA did not improve on average, but rather decreased in two examples [8]. However, SCAs are very diverse. One study compared the accuracy of two SCAs in diagnosing inflammatory rheumatic diseases with physician diagnosis and concluded that the diagnostic accuracy of SCAs is limited [9]. A study by Fraser et al. investigated diagnostic and triage accuracy of a symptom checker used by patients at an emergency department [10]. The symptom checker’s diagnoses were compared with those of emergency physicians and the final diagnoses from the emergency department. This study concluded that SCAs can provide acceptable diagnostic accuracy for patients with various urgent conditions [10]. One study by Gilbert et al. compared the extent of disease coverage, the accuracy of the suggested diseases, and the appropriateness of the recommendations from diverse SCAs with general practitioners (GPs) [11]. In this study no SCA outperformed the GPs. Although SCAs promise to improve diagnostic processes, reduce misdiagnosis, and guide patients more effectively through health care systems, there exists little supporting evidence at the moment.

In addition, the motivations why people use SCAs and whether they find them useful has been little studied. Furthermore, it is unclear if people use SCAs to supplement medical advice or as a substitute for in-person physicians’ visits. A pilot study by Miller et al. examined the usability, acceptability, and utility of an SCA in primary care [12]. In this study, the symptom checker was evaluated as very user-friendly and acceptable by patients in primary care [12]. One study by Meyer et al. examined patients’ experiences using an online symptom checker [13]. Despite ongoing concerns and little empirical evidence about the accuracy of SCAs, patients in the study by Meyer et al. perceived the symptom checker as a useful tool for diagnosis [13]. Further studies investigated the perspectives on symptom checkers by young adults [14] or older adults [15], and in specific contexts, for example, in cases of rheumatology [9; 16] or at emergency departments [10]. It is crucial to know and understand the users’ perspectives on SCA use, as these insights can show how SCAs can be improved and implemented in a responsible way.

There are sporadic debates on the ethical and social aspects of SCAs. It is debated, for example, who should have access to this new technology, whether these apps might empower users or whether they are an acceptable substitute for a medical consultation. It is also debated whether SCAs lead to over- or under-triage and whether (legal) regulation is needed [6]. While the ethical debate about SCA might be sporadic, there is a growing ethical debate surrounding mHealth and Health Apps in general [17–20], and it should be remembered that SCAs are situated within this context. Although a wide variety of perspectives play a role in these ethical debates, one of the most important perspectives, that of the user, remains underrepresented. The discussions on the ethical implications of SCAs are often poorly supported by empirical data. Furthermore, the subjective perspective of the users and the ethical aspects are not interlinked yet.

This qualitative study investigates the subjective perspectives of SCA users to address the ethical and social aspects in the context of SCA use. The aim is to investigate the subjective perspectives of SCA users to bridge the empirical gap in the existing literature by answering the following main research question: How do lay persons (healthy users and patients) experience the usage of SCAs, including their attitudes, expectations, motivations and concerns regarding their SCA use? The study focuses on SCAs that are for lay persons (healthy users and patients), not for healthcare professionals, and do not focus exclusively on specific types of illness.

## Methods

### Study design

This qualitative study is part of the project CHECK.APP [21]. This project investigates the impact of SCAs at different levels of the healthcare system (at the micro, meso, and macro level), from different disciplines (ethics, law, sociology, social medicine, occupational health medicine, and health services research), and from multi perspectives ((non)users, general practitioners, and healthcare experts) [21]. The CHECK.APP project has different, but overlapping study phases, starting with a comprehensive literature review on the ethical, legal, and social aspects of SCAs [6], a representative survey on the usage of SCAs in Germany, a self-observational diary study combined with qualitative user interviews, and lastly, qualitative interviews with GPs and healthcare experts. This paper focuses particularly on the data from the individual user interviews, highlighting users’ experiences, expectations, and assessments.

The results of the preceding study phases (review, survey, and diary study) influenced the objectives and design of the qualitative interview study. Based on the empirical research gap - identified by the scoping review [6] - an explorative study approach was chosen. The lack of empirical data on the subjective user perspectives in the literature on SCAs informed the research objective: to gain a first-hand understanding of those using SCAs. Accordingly, an explorative qualitative design, namely a qualitative semi-structured interview-study, was chosen to clarify the relatively unknown experience of SCA use.

The main research question is, how do individuals using SCAs experience their usage. This overarching question is divided into five sub-questions: What are the users’ expectations of SCAs? How do users assess the findings and recommendations of the SCA? How do the users perceive the communication with the app? How do they perceive the role of the app in the context of a physician’s visit? What risks do users notice when using SCAs? These sub-questions were derived through the research within the CHECK.APP project, i.e., from previous studies and regular discussions within the research group. According to these five sub-questions, the framework for the interview study and the following analysis were structured around five themes: (i) users’ expectations regarding SCA use; (ii) assessment of findings and recommendations; (iii) communication; (iv) role of the app in the context of a physician’s visit; (v) risks.

Based on these five themes, a semi-structured qualitative interview guide was developed in a stepwise process. In the first step, using the results of the literature review and the precedent survey, tentative questions for the interviews were formulated. In particular, the participants’ notes of the diary study were used as a basis for developing the interview questions. In the second step, all questions collected were checked for their suitability, e.g., whether the questions were relevant regarding the objectives of the study and the main research question. In the last step, the relevant questions were sorted and grouped according to the five themes. The resulting interview guide had around 10 questions, and a planned duration of about an hour. The guide starts with open-ended, more narrative questions and ends with more specific questions on normative issues (see Supplement 1: Interview Guide English). Two pilot tests were conducted in December 2021. These two pilot interviews were included in the final analysis as they resulted only in minor modifications to the interview guide. The final semi-structured qualitative interview guide was then applied for interviews with SCA users.

### Sample and recruitment

The CHECK.APP project used different recruiting tools to get a representative sample. For the first project phase, German citizens were contacted via mail by an external partner to participate in the representative survey. Additional recruiting was conducted by mailing lists of the University Medicine Tübingen, and social media. Inclusion criteria for participation in the CHECK.APP project was the ability to give consent and German language skills of at least B1 of the Common European Framework of References for Languages.

For the diary study the sample was then restricted to participants who had previous experience with SCAs, specifically with the symptom checker Ada [22]. Ada was used in the qualitative parts of the study as an example of a SCA, as it is one of the most popular SCA in Germany. The participants for the individual interviews were recruited from the previous diary study. The content of the diary-based self-observation, SCA usage behaviour, medical indication, and socioeconomic factors were used as criteria for sampling. The sample size was calculated on the 5D model of information power by Malterud et al. [23].

All study participants were informed orally and in written form about the research goals of the CHECK.APP project, the process of the study (parts) and their rights. The participants got financial compensation for participating in the individual interviews and a following member check.

### Data collection and analysis

Due to the COVID-19 pandemic, the semi-structured qualitative interviews were carried out via video call (Zoom Meetings). The interview study was conducted by two male and two female researchers (RM, MK, RK, and one assistant), who worked in the CHECK.APP project at the time of the study and are trained in qualitative methods. The interviewers had different credentials (PhD and MD) and various disciplinary backgrounds (Medicine, Philosophy/Ethics, and Social Sciences). Each interview was led by two researchers from different disciplines in tandem. While one researcher asked the questions, the other researcher took notes. The researchers discussed the interviews after their completion and in regular project meetings. All interviews were audio-recorded and transcribed verbatim by an external partner.

The interview transcripts were randomly distributed to three researchers (RM, MK, and one assistant) who analysed the transcripts by content analytical procedures. The methodology selected for the data analysis was qualitative content analysis according to Mayring [24]. This data analysis technique provides a systematic way of reducing and synthesising a wide range of data. Its central idea is to assign categories to text passages through a qualitative-interpretative act. The technique follows a systematic procedure and strict content-analytical rules combining deductive and inductive category development. The aim was to shorten the large data material and filter the essential contents via reduction and progressive generalisation.

The three researchers worked through the interview transcripts with the aid of the software program MAXQDA and with the previously developed, deductively obtained system of the five themes: (i) users’ expectations regarding SCA use; (ii) assessment of findings and recommendations; (iii) communication; (iv) role of the app in the context of a physician’s visit; (v) risks. Before starting the investigation, the units of analysis and the level of reduction and generalisation were defined. In the first step, the researchers independently formulated text passages relevant to the research questions into simplified short paraphrases (paraphrasing). In the second step, the contents of the paraphrased passages were generalised on the previously defined abstraction level (generalisation). In the third step, the generalised passages were reduced and summarised into central categories (reduction). While the researchers discussed preliminary results, uncertainties and emerging questions during the coding process, each researcher individually worked through the transcripts. In the last step all developed categories were summarised by one researcher (RM) into a final category system, which was then back-checked on the original material and through discussions among the CHECK.APP researchers.

Coder influence was controlled by three researchers (RM, MK, and one assistant) with different professional backgrounds being involved in the data interpretation and repeating discussions in the CHECK.APP project during the analysis. Additionally, in the middle of the analyses a digital member check was conducted with participants of the CHECK.APP study to test the validity of the results. During the member check, we presented our interim results to the interview partners and asked them for their feedback on these results. The member check was seen as a tool to validate partial results of the interview analysis while checking the relevance of the results for the people concerned. Internal methods-workshops in the CHECK.APP project and an external advisory board served as further tools for quality control.

The study was conducted in accordance with the Declaration of Helsinki [25]. Ethical approval was obtained from the ethics committee of the University Tübingen. Ethics research requirements, such as informed consent and data protection, were carefully considered. The reporting of this qualitative study is oriented on the COREQ checklist for reporting qualitative studies [26].

## Results

Fifteen interviews were conducted between January and March 2022 in Germany via video call. The interviews lasted an average of 46 min, ranging from 35 to 76 min (median 41 min). The age of the participants ranges from 20 to 69 years, yet the most participants were between 20 and 29 years. Nine interviews were conducted with male SCA users, six interviews with female users. The recruiting tools did not lead to the inclusion of trans- or intersex-persons in the sample. Most of the participants are well educated (‘Abitur’) and live in rural areas. The participants’ characteristics can be seen in Table 1.

**Table 1:** Participants’ Characteristics

Characteristics		N
**Total participants**		15
**Gender**		
	Male	9
	Female	6
	Diverse	0
**Age**		
	20–29	10
	30–39	2
	40–49	1
	50–59	1
	60–69	1
**Level of Education**		
	Higher School	12
	Secondary School	2
	Main School	1
**Place of Residence**		
	Rural	13
	City	2

We analysed all fifteen interviews. Throughout the analysis the framework with the five predefined themes was modified through the coding process and expanded to seven categories (in alphabetical order): (1) Attitudes towards findings and recommendations, (2) Communication, (3) Contact with physicians, (4) Expectations (prior to use), (5) Motivations, (6) Risks, and (7) SCA-use for others. The distinction between attitudes, expectations and motivations had been developed deductively. The differences, for example, between an attitude and a motivation were not easily discernible in the interview material. Expectations can be understood as a transverse category, because they are implicitly reflected in all categories. Nevertheless, we were explicitly interested in the users’ expectations prior to their SCA use, as opposed to their attitudes after their SCA use. Expectations, in our understanding, refer to the app (e.g., “The SCA should do this or that…”). By motivation, we understood statements that refer to the users themselves (e.g., “I use SCA to….“).

During the process of categorisation, up to five levels of subcategories were identified and 1052 codes were assigned all up. Table 2 shows all categories and subcategories up to the fifth level, and the frequency of their overall occurrence. Selected study results will be presented in the following sections according to the seven main themes and, subsequently, selected aspects will be discussed.

**Table 2:** Categories and Subcategories

List of Codes	Frequency
Code system	1052
**Attitudes towards findings and recommendations**	**0**
**Adherence (to recommendations)**	**0**
Following	29
Not-following	29
**Advantages of SCA use**	**0**
Confirmation/reassurance	24
Decision support regarding further actions	16
Expertise/seriousness	14
Frequencies	8
Information/Orientation	10
Naming Causes	3
Overall satisfaction/confidence	53
Reliability	2
Timeliness of the query	1
Useful for reminder/documentation	2
**Criteria for assessing findings**	**0**
Adding other sources of information	8
Comparison with family history	1
Medical verdict	4
Perceived plausibility	2
Reliance on your own abilities	11
**Disadvantages of SCA use (as compared to other sources)**	**0**
Bias through users	8
Criticism regarding the findings	0
Existing diagnosis not recognized	8
Implausible	8
One-sided	2
Functional logic of SCA	0
Intransparency	2
Networking	1
Risk averse	8
General criticism	8
Mental stress	20
No information on self-treatment options	5
No objectivity/reliability	4
No trust in findings	8
Probabilities aren’t helpful	4
Room for interpretation	12
Unspecific	6
**Effects on users’ perceptions**	**4**
**(No) perception of findings as a diagnosis**	**24**
**Personal factors influencing assessment**	**2**
Influence of personality traits	10
Hypochondria	4
Symptoms with a history	5
**Communication**	**1**
**Advantages**	**5**
Affinity for technology	1
Dealing with uncomfortable topics	5
Detail	17
Feeling of being noticed	1
Illustration/localization of symptoms	3
Impartiality/Openness	2
Similar to human dialogue	5
Simplicity	7
**Comparisons of communication with a physician**	**30**
**Disadvantages**	**0**
Limitations	2
No real dialogue	4
Not human or irritation caused by antromorphization	9
Technical terms/text load	10
Tediousness	4
Uncertainties	5
Unfocused	4
**Mode of communication**	**0**
Playful	5
Truthful	17
**Contact with physicians**	**0**
**Comparison SCA - physician**	**0**
Advantages of the physician’s visit	2
Personal/individual/comprehensive view	5
Professionalism	1
Prognosis	1
Recipe exhibition	2
Relevance of physical examinations	9
Reliable diagnosis	1
Subsequent treatment	2
Benefits of SCA	1
App cannot reject	2
Detail	3
Holistic view	1
Objectivity	1
Practicability	1
SCA is not a substitute for a physician	19
Trustworthiness	20
**Decisions regarding medical consultation**	**20**
Own classification	4
Reasons for physician contact	1
Acute/unknown symptoms	6
Confirmation/clarification	2
General reasons	5
Notification of illness	1
Suffering/severity of symptoms	15
SCA as a decision-making aid in advance	6
**Role of SCA at physician’s visit**	**0**
Confirmation of SCA results	3
Discrepancy in results	4
Documentation/reminder	3
Influence on the relationship	1
Mention to physicians	0
Barriers	8
Mention	12
Astonishment	2
Benefits of Mentioning SCA	8
Desired response: discourse and collaboration	2
Negative Reactions	5
Positive Reactions	6
No mention	0
“Concealment” of the app	4
Negative reactions expected	5
Stay abroad	1
The physician does her own exam anyway	1
No influence	2
Review afterwards	1
SCA as a supplement	2
Use in advance/preparation	8
Wishes for cooperation	4
**SCA as recommendation**	**5**
**Expectations (prior to use)**	**108**
**Adjustment of expectations (through SCA use)**	**1**
**Aggregation of symptoms**	**2**
**Backup**	**1**
**Confirmation/reassurance**	**16**
**“False” expectations (from other users)**	**0**
Findings as diagnosis	2
General scepticism	2
Limits	12
Results not ultimately valid	3
**First aid/orientation**	**27**
Assessment of the risk	8
Decision support: physician contact	3
Early detection/prevention	1
**Helpful with little things**	**1**
**Knowledge/expertise**	**16**
**Little or no use**	**13**
**Overcoming barriers**	**1**
**Savings**	**9**
**Showing alternatives**	**2**
**Motivations**	**0**
**Apprehension**	**1**
**Avoid physician visits**	**0**
Avoid physician visits abroad	4
Avoid unnecessary physician visits	3
**Benign diseases**	**5**
**Curiosity/interest**	**39**
**“Testing“ SCAs**	**14**
**Risks**	**0**
**No concerns**	**32**
**Subsequent assignment of responsibilities**	**27**
To SCA	2
To app users	24
**Concerns**	**0**
Incorrect findings (and following incorrect behaviour)	4
Mental distress	5
Missing aspects	3
Non-critical use	5
Over-triage	8
Privacy	3
Self-treatment	3
Symptom intensification	3
Under-triage	10
**Sense of security**	**14**
**SCA-use for others**	**18**
**Addressee**	**0**
Family (children, partner)	7
Technology-critical people	2
**Problems**	**0**
Privacy	2
Responsibility	2
**Unproblematic**	**5**

### Attitudes towards findings and recommendations

The study revealed that those using SCAs assess the findings (in the following we use “findings” and “symptom analysis” synonymously) and triage-advice provided by the app (in the following “recommendations”) very heterogeneously, depending, for example, on their chosen criteria for assessment. This first main category “Attitudes towards findings and recommendations” encompasses seven subcategories (in alphabetical order): 1.a) Adherence (to recommendations), 1.b) Advantages of SCA use, 1.c) Criteria for assessing findings, 1.d) Disadvantages of SCA use (as compared to other sources), 1.e) Effects on users’ perceptions, 1.f) (No) perception of findings as a diagnosis, 1.g) Personal factors influencing assessment.

Regarding the subcategory 1.a) *Adherence (to recommendations)* the interview participants described both behaviours: following or not following the app’s advice. Whether the recommendations were followed (or not) depended on the given symptoms, the listed diseases, one’s own experience (e.g., family history of the disease), the assumed probability that the findings are correct, and one’s own assessment. Reasons for following the recommendations were personal reasons (such as time resources), individual suffering (e.g., current pain), urgency and severity of the listed diseases, plausibility of the SCA results, and fear (e.g., of worsening symptoms). Reasons for not following the recommendations were also personal reasons, including long waiting times for physical appointments, no confidence in the SCA results, a “wait and see” attitude, perceived implausibility, incorrectness, and unspecificity of the SCA results. No acuteness and no emergency cases were also stated as reasons for not following the SCA recommendations. For example, one participant said that she would have disregarded the app’s recommendation had she been suffering from tonsillitis. The reason she gave was that tonsillitis, not being life-threatening in her view, did not warrant a visit to a physician or an emergency room. Consequently, she cited the lack of necessity as her reason for not following the app’s advice.

*“In the case of tonsillitis, it is nothing life-threatening. I then have pain in my throat that most likely needs to be treated with antibiotics, but I don’t need to see a doctor or someone who treats me acutely or initiates any emergency measures that I would urgently need in the event of a stroke or heart attack or something. And that’s why I didn’t follow the app’s recommendation for the tonsillitis, because I knew it wasn’t necessary.” (NT 39)*

Regardless of whether the recommendations were followed or not, they were considered primarily as an orientation or guidance by the SCA users.

In the interviews, several positive aspects of using SCAs were mentioned, which we summarised under subcategory 1.b) *Advantages of SCA use*. Here, confirmation and reassurance through app use were described by the participants. The support via the SCA regarding decisions and further actions was valued. The expertise and seriousness of SCA were emphasised, in particular compared to simple googling. Furthermore, information, orientation, and the naming and frequencies of possible causes were highlighted as further positive aspects. Some participants described a general satisfaction and confidence with the SCA results and emphasised their reliability. The timeliness of the query and its usefulness as a reminder and for documentation were also described.

In the subcategory 1.c) *Criteria for assessing findings* we collected the criteria that users relied on to assess the list with the possible causes. Most participants reported that they did not only rely on the list and information provided by SCA but turned also to other sources of information. Other digital search tools, such as Google, other apps and third parties, such as friends or family members, were mentioned. One participant reported, for example, that she compared the findings of SCA with her own family medical history. In addition, many participants reported that they asked for further verification and specification by physicians to confirm (or reject) the symptom analysis of the SCA. For example, one participant regarded the findings of the SCA as a kind of recommendation that should be clarified with a physician, especially in urgent cases. In the user’s assessment of the findings, the physician would provide more certainty than the SCA.

*“[…] with the app, it’s more, I would say, like a recommendation for a diagnosis. So, if it were something really serious, I would definitely go to the doctor afterwards and clarify it with him, maybe also show him what the app said. Then he’ll probably make his own medical checks, but I think that in the end, it’s to a certain extent the same, but I wouldn’t put the same certainty [on the app results], as when I go to an expert.” (NT 43)*

The plausibility of the findings provided by the SCA was an important factor for the users in their assessment of the app. Participants were more likely to accept results that seemed plausible to them, but were sceptical about the app’s findings and recommendations if the listed causes or conditions were unknown to them or had no connection to their individual experience. Consistency with their knowledge, personal experience and the current individual situation served as further criteria for the plausibility of SCA findings.

*“If you had received a diagnosis that was completely different from anything you had heard before, I probably would have used other online tools first and looked for diagnoses to see if something like that even made sense or why it came up. Whenever something came up that was plausible to me, I took it seriously […] if it was consistent. My personal attitude: sceptical towards recommendations if they don’t match other things, or if you’ve never heard them before. Then online search or in other apps, whether this makes any sense.” (NT 47)*

Another criterion for users in assessing the app was their own (body) sensation. The interview participants emphasised their abilities and feelings to assess the SCA’s findings. One participant, for example, said that he “would put [his] body feeling above the app results” (NT 21). Awareness of one’s own body, trust in one’s own abilities, common sense, and gut feelings were referred by the participants as criteria assessing the SCA’s findings.

All interview passages in which the app’s symptom analysis and recommendations were negatively described were summarised in the subcategory 1.d) *Disadvantages of SCA use (as compared to other sources).* Some participants expressed concerns that the SCA findings could be negatively influenced and biased by their own user behaviour. One participant, for example, reported that he tried to use the SCA with utmost neutrality. He aimed to avoid letting his prior knowledge to sway the questions and, therefore, the outcome of the SCA.

*“I tried to use the app as neutrally or as unbiased as possible, even if I already had something in my mind. Nevertheless, I tried to answer the questions as they come without being influenced by it, simply because, with my previous knowledge, I don’t want to steer the questions in a certain direction that I like, perhaps, or the way I think it might be.” (NT 44)*

Like this participant, many users worried that their inputs could inadvertently influence or “determine” the questions from the chatbot and, accordingly, the findings. SCA users’ emotions, assumptions, and incorrect knowledge were seen as aspects that could lead to incorrect input. According to the users, instead of neutral input, the users could (more or less unconsciously) influence the SCA’s final findings by their answers and choices. In addition to this concern, the participants also directly criticised the SCA’s findings. It was criticised that already known diagnoses were not recognized by the SCA. Concrete examples were migraine, asiderosis, and diverse skin rashes. Further critiques described the SCA’s findings as implausible or one-sided. The participants also complained about the functional logic of the SCA, specifically about the intransparency of the SCA. For example, when they could not understand the order of the questions in the chat. Some recommendations of the app were evaluated as too extreme (e.g., going to the emergency room). Some participants referred in this context to the risk-aversion inherent in the SCA. Another disadvantage was seen in the “stand-alone-character” of the SCA, e.g., that the SCA is not connected with other information services or tools. Further critiques were an increase of mental stress, the lack of further information on self-treatment options, no objectivity or reliability of the apps’ findings, and therefore no trust in it. Some participants found the results to be too nonspecific and the probabilities displayed to be unhelpful, leaving too much room for interpretation.

Some participants also reported effects on their own (body) perceptions through SCA use. These effects were summarised in the subcategory 1.e. *Effects on users’ perceptions*. One participant described being more aware of his own body and being more reflective about his body as a result of SCA use. Another participant said that the use of the app brought some aspects to the forefront that she would not have otherwise paid attention to. In contrast, some participants described that SCA use had no effect on their perception of their own body feelings or symptoms.

In the interviews, there were different statements about whether the SCA findings could be understood as a diagnosis or not. We summarised these statements in the subcategory 1.f) *(No) perception of findings as a diagnosis*. Some participants called the app’s findings a diagnosis, saying it would be the same or at least equivalent to a medical diagnosis. In contrast, other participants stressed that the SCA findings could not be understood as diagnoses and emphasised the differences compared with both their own assessment and a physician’s diagnosis. One participant described, for example, *“It was not a diagnosis for me because it did not match my feelings […] I wouldn’t take it seriously without asking a doctor.”* (NT39) The participants expressed opposing views and a lot of uncertainties on that topic. Often, they had no clear opinion and described it rather as gradual distinction or continuum (e.g., *“It is not a complete diagnosis”* (NT31), *“to some extent it is a diagnosis”* (NT19)). Whether the SCA’s findings were perceived as a diagnosis depended on various aspects, for example on the severity of the symptoms and urgency of the treatment. The participants referred to the physician’s judgement, the plausibility of the results, their own abilities, and other sources of information. Very often the participants perceived the app’s findings as a hint or an impulse to the “correct” diagnosis, whereas the “correct” diagnosis was defined by a diagnosis made by a physician. One participant stated for example: *“The SCA gives me an impulse to say, maybe it could go in that direction or that. But at the doctor’s, it’s just that I expect a very specific correct diagnosis.”* (NT21) The judgement by a physician and the specificity of this judgement were seen as prerequisites for a “correct” diagnosis. In addition, the physician’s diagnosis was often assumed to have a certain authority, that was not doubted. For example, one participant described the diagnosis as something conclusive. *“For me a diagnosis is such a final thing, so when someone goes to the doctor and gets a diagnosis, you don’t doubt it, that’s just the way it is.” (NT43)* Aspects for a complete or correct diagnosis were the inclusion of other examinations (e.g., physical examination or laboratory procedures) and following steps (e.g., treatment options, referrals to specialists, or medical prescriptions). The participants emphasised that both cannot be provided by the SCA.

The participants described the influence of personal factors on their app assessment. These statements were summarised in the subcategory 1.g) *Personal factors influencing assessment*. The participants explained that personal traits, such as a timid character, can influence how the users assess the app’s findings and response accordingly. An example often given in the interviews was that an anxious person would evaluate the app’s findings differently than a risk-taking person and, accordingly, behave differently. The term “hypochondria” has often come up in this context. Another example was that the personal patient history can also have an impact on how individual users respond to the app’s findings and recommendations.

### Communication

In the second main category “Communication” we collected all interview passages that referred to the communication of the SCA users. This covers the interaction between users and their SCA, but also how the SCA influenced the users’ communication with others, for example with physicians. This main category has four subcategories (alphabetical order): 2.a) Advantages, 2.b) Comparison of communication with a physician, 2.c) Disadvantages, and 2.d) Modes of communication.

We subcategorized all positive aspects described by the participants when asked about their communication with the SCA in the subcategory 2.a) *Advantages.* In some interviews a correlation between a good communication with the SCA and a general affinity for technology was stated. It was reported by the participants that SCA made it easier to deal with unpleasant topics, for example body weight or sexually transmitted diseases. In addition, it was emphasised that the SCA went into detail and could visualise or localise symptoms, for example on an image of the human body in the app. The participants reported that the SCA was impartial and open, and simple to use. Some participants perceived the chat as similar to a human dialogue and felt “noticed” by the app. When describing how the SCA can affect their communication with physicians, participants reported positive as well as negative aspects.

*“[…] when I have symptoms, I always look on the internet, in the app or somewhere else. And then I have an idea, what it could be. And then, of course, I go to the doctor with a certain expectation and see if this is confirmed. So, I think that [the SCA] has an influence on the conversation with the doctor because I emphasise certain symptoms more than others. And I can describe my health more accurately than if I hadn’t searched before. Maybe that’s good in some cases because it leads to a quicker result. But maybe it is also negative because I lead the doctor on a wrong track […].” (NT 06)*

Like this SCA user, several participants described that using SCA could lead to the development of expectations that could influence their interaction with physicians. The positive effects would be, on the one hand, better information and a detailed description of the patient’s symptoms. On the other hand, a potential negative effect could be the unintentional influence on the physician’s judgement.

Many participants compared their communication with the SCA to their communication with a physician. These statements were summarised in the subcategory 2.b) *Comparisons of communication with a physician*. The participants described their dialogues with physicians as more personal and interactive: physicians would listen to them, take their needs seriously, respond to what they say and ask further questions. Furthermore, physicians can recognize facial expressions and gestures and question what is said by the users. In contrast, the communication with the SCA was not described as a dialogue, but rather as impersonal or non-human because of its “yes” or “no” questions.

*“If you ask me whether it feels as if you’re in a dialogue, then I have to say quite clearly: No. It’s just such a sequence of yes and no questions, which of course also restricts the diagnostic capability, I would say, but, yes, … I think we’re technologically not advanced enough yet to make it better or convincingly human.” (NT 24)*

At the same time, the communication with the SCA was reported as rich in details, more comprehensive, and more neutral. In particular, the interview style of the app was perceived as positive by some participants.

*“I think this interview process is quite good. In part, you feel very well perceived, simply because of the questions. And if you compare that with a doctor, well, maybe a doctor, maybe a family doctor knows you. Maybe he knows more about you. But otherwise, very detailed questions are asked by the app, which a doctor would probably not ask. At least that’s how it seems to me.” (NT 07)*

The many detailed questions of the SCA were described as positive in comparison to physicians, as they would, among other things, help the SCA user to feel perceived. Despite these positive aspects, various negative aspects regarding the communication with the SCA were reported. These statements were categorised in the subcategory 2.c) *Disadvantages*. In addition to general limitations of communication with an app, the participants criticised that there is no human counterpart and thus no real dialog with the app or chatbot. Some participants were irritated by anthropomorphizing features of the chatbot and explained personal uncertainties in this context.

*“Sometimes I think it’s a little bit too much, as the app is trying to replicate a person or a real diagnostic conversation, I’d say. Because sometimes, it’s like you would say something in a conversation. I always find that a bit strange with apps, because it’s just an algorithm that calculates something. There’s no one, but it doesn’t really bother me. I think it just seems a bit more friendly. People are probably more willing to answer questions.” (NT 13)*

While some users found the SCAs’ attempt to mimic personal conversation slightly disconcerting, it did not discourage future use. Some saw the purpose of this imitation as motivating the SCA users to answer the questions of the app. Some participants perceived the communication with the SCA as too technical and text loaded. The chat’s tediousness and lack of focus were criticised. Some participants spoke of the SCA as a gimmick and described their playful mode of interaction with the app. Most participants reported a truthful communication with the app, meaning that they entered truthful information about themselves (e.g., age) and their symptoms in the app.

*“I found it quite practical, because things are asked that would perhaps be a bit more unpleasant to answer, at first, if a person would ask them to me. With the app, I’m sitting in front of it alone and can simply choose. That’s why it was easier for me to answer more truthfully.” (NT 42)*

In some cases, as in this quote, it was described as easier to answer the SCA’s questions truthfully than questions from a person, especially when it came to unpleasant topics. Being alone with the app seems to facilitate a greater level of honesty among some users.

### Contact with physicians

The third main category “Contact with Physicians’’ includes all interview passages concerning contact or a link between the SCA users and physicians. This main category has four subcategories: 3.a) Comparison SCA – physician, 3.b) Decisions regarding medical consultation, 3.c) Role of SCA at physicians visit, and 3.d) SCA as recommendation.

In the subcategory 3.a) *Comparison SCA – physician* we collected all statements in which SCAs were compared with physicians. In this category, advantages of the physician’s visits were described, such as the personal, individual, and comprehensive view of physicians. Most participants considered the physicians as professionals. In their view, and in contrast to SCAs, physicians could give reliable diagnosis, prognosis, and prescriptions. In addition, they could recommend subsequent treatment options.

*“Well, the doctor is of course professional, he gives more individual information. Especially the family doctor, he knows me. He also knows if there are any pre-existing diseases. And he can of course give a more precise diagnosis, or more precise information about what it could actually be. And above all, how do we treat it? Of course, I can only find that out from my doctor, I didn’t find that out from the SCA. […] in principle I would say that [the SCA] definitely does not replace a visit to the doctor.” (NT 19)*

The participants emphasised the relevance of the physical examination and underlined that an app cannot provide such an examination. In contrast, the participants also pointed out advantages of SCA usage. Some participants highlighted that the SCA could not reject a question or request, compared for example with long waiting times for a physician visit. Other participants highlighted that the SCA could ask in more detail and had a more comprehensive view. Still others emphasised that the SCA was more objective and practical. Even though a lot of advantages were addressed, the participants emphasised that the SCA was not a substitute for medical care from a trained and licensed physician. In this context, the trustworthiness of physicians and the SCA played a role.

In the subcategory 3.b) *Decisions regarding medical consultation* we collected all statements from the participants deliberating about using a medical consultation. The participants mentioned various reasons for getting in contact with physicians. Besides their own assessment, acute, very severe, and unknown symptoms as well as suffering were occasions for them to consult a physician. The confirmation or clarification of the symptoms through medical professionals and the notification of diseases were further reasons.

*“For example, if I now had some symptoms, which really concerns me very strongly, where I now would have thought to myself: Hmm, maybe it would be better now [to go to the doctor]. And then I would have looked in the app and the app would confirm me in this thought, so then the visit to the doctor would be worthwhile.” (NT 37)*

Some participants saw the SCA as confirmation of their intention to see a doctor. In general, the participants saw the SCA as a decision-making aid prior to medical consultation.

The third subcategory 3.c) *Role of SCAs at physician visits* includes all interview passages concerning the role of the SCA at physicians’ visits. Various roles were stated throughout the interviews. Many participants regarded the SCA as a useful reminder or tool for the documentation of their symptoms. The participants described both the confirmation of SCA results by physicians, but also discrepancies between SCA and physicians. In this context, the influence of SCA on the user-physician-relationship and the reference to the SCA during the physicians’ consultation were addressed by the participants. Some participants described difficulties mentioning the SCA to physicians, while others expressed no concerns. In the case of mentioning, some participants reported a lack of knowledge and astonishment as a reaction from the medical professionals.

*“He [the doctor] found it very good that I had already used this app. But he didn’t know about it until then. I don’t know if he looked at it once at some point. He was quite surprised that something like that exists. […] he said: Yes, that’s just future medicine. But in any case, it didn’t have a negative influence. Whether it was positive? I would simply say it was neutral. He thought it was good that I read through the app. Of course, he also said that it will not replace his diagnosis. But, yes. It was in no way negative.” (NT 39)*

In general, the participants experienced both positive and negative reactions. When asked what reactions they would wish for, the participants mentioned more discourse and collaboration with the medical professionals. In case of differences between what the physician says and what the SCA outputs, many participants would trust the physician more than the SCA. Knowledge of each other plays a role in this assessment and again the unquestionable nature of the information provided by the physician was mentioned.

*“Assuming there is a difference between what the doctor tells me and what the SCA tells me, I would ask more questions based on the information I have from the SCA. […] So I guess my trust in the doctor is relatively high. I’ve known him for a relatively long time and so on. So, I don’t know, I would definitely ask [the doctor], maybe something will come out of it. But I think if it were different, I probably wouldn’t doubt it [….].“ (NT 04)*

A few participants said that they would not mention or even conceal their SCA use to the physicians. Reasons for hiding the SCA were expected negative reactions and different treatment. One participant described, for example, his concern that the physician would treat them differently, as soon as she found out about his SCA use.

*“I haven’t mentioned to any doctor that I’ve already tried this with the app […] because I assume that the doctor then deals with the patient in a completely different way. Well, not every doctor, but maybe some. Because he then [would say]*

* “If you already ask Doctor Google, why then you come to me, then you know that already better than I do”*.

*I don’t want to give that impression, I simply let the doctor do. […] but, as I said, I didn’t mention the use of the app anywhere, not to any doctor.” (NT 19)*

Other concerns included rejection and having to justify the visit to the physician. To prevent this, the participants would conceal their SCA use. Another reason for hiding their SCA use was the irrelevance of the SCA usage. Meaning, for example, that the physicians would do their own medical checks irrelevant from the SCA results.

Some participants used the SCA in advance, for example to prepare a visit. Others used it afterwards, to review or check the visits’ results. A number of participants interpreted the SCA just as a supplement to the physician’s consultation. However, regardless of the specific use, the participants wished for more cooperation between users/patients and medical professionals in this context. A few participants used the SCA because it was recommended to them by medical professionals. These statements were collected in the subcategory 3.d) *SCA as recommendation*.

### Expectations (prior to use)

The main category “Expectations (prior to use)” covers all interview statements in which the participants described their expectations before their SCA use. This main category has twelve subcategories: 4.a) Adjustment of expectations (through SCA use), 4.b) Aggregation of symptoms, 4.c) Backup, 4.d) Confirmation/reassurance, 4.e) “False” expectations (from other users), 4.f) First aid/orientation, 4.g) Helpful with little things, 4.h) Knowledge/expertise, 4.i) Little or no use, 4.j) Overcoming barriers, 4.k) Savings, and 4.l) Showing alternatives.

The subcategory 4.a) *Adjustment of expectations (through SCA use)* covers the participants’ idea that SCA users need to adjust their expectations to what the SCA could actually deliver. Besides that, the participants described various expectations. One expectation is that the SCA could aggregate several (trivial) symptoms over a long period to one concrete diagnosis. Statements in this direction were summarised in the subcategory 4.b) *Aggregation of symptoms*. Another expectation is that the SCA was a backup option when, for example, doctor’s offices are closed or the user’s family doctor is on vacation. These expectations were collected in the subcategory 4.c) *Backup*. The subcategory 4.d) *Confirmation/reassurance* includes all text passages in which the participants described the confirmation of their previously held assumptions as one of their expectations.

Several participants spoke not about their own expectations, but about “false” expectations from other users. For example, some participants were concerned that other users might mistakenly expect a diagnosis from the SCA. Because of that concern, the participants argued for a general scepticism toward the SCA, indicating the limits of SCAs. These worries were grouped into the subcategory 4.e) *“False” expectations (from other users)*. By contrast, first aid and orientation were appropriate expectations from the perspective of many participants, collected in the subcategory 4.f) *First aid/orientation*. Concrete expectations in this subcategory were the assessment of the actual risk (“It’s just a safeguard.” (NT 44)), decision-support prior to medical contact, and early detection and prevention of diseases. Expectations regarding trivial symptoms were summarised in the subcategory 4.g) *Helpful with little things*. This subcategory covers the expectation that SCAs could help the user especially with minor symptoms. Another expectation was precise information and expert knowledge, in particular comparing SCAs with internet research tools such as Google. These expectations were summarised in the subcategory 4.h) *Knowledge/expertise*. Despite the expectations of precise information and specialist knowledge, the SCAs were at the same time differentiated from physicians. One participant stated, for example, that she did not expect the SCA to make decisions or replace a physician’s appointment.

*“I didn’t use the app expecting it to have the last word. In other words, I didn’t expect it to be able to replace a visit to the doctor if things were really serious.” (NT 06)*

A few participants denied having expectations before their SCA usage. They reported no SCA use in their daily lives because they had no symptoms or complaints and, to that extent, no need for it. Others reported that they had symptoms but were already aware of them or the underlying disease. Still others had very low expectations and a general scepticism as to whether SCA can work at all. In this context, one participant referred to personal contact with the physician:

*“I think my expectations were so low because I was thinking: This can’t work. How should this replace a personal medical assessment? Of course, it can’t do that, because of course you also have a doctor with whom you can talk a bit more, also with regard to the treatment afterwards. The app doesn’t do that, nor should it.” (NT 39)*

Another expectation was that SCAs could help anxious or shy people to go into contact with medical professionals. These expectations were collected in the subcategory 4.j) *Overcoming barriers*. The subcategory 4.k) *Savings* summarises all expectations that SCA usage could lead to personal savings. Here, examples were long ways to physicians and long waiting times. The subcategory 4.l) *Showing alternatives* collects all statements of the participants that SCAs might show further possibilities and alternatives to already existing knowledge and findings (“type it in again, maybe something else will come up” (NT 31)).

### Motivations

The main category “Motivations” was one of the categories directly delivered from the interview material and covers all interview statements concerning the motivation of the participants to use SCAs. This category has five subcategories (alphabetical order): 5.a) Apprehension, 5.b) Avoid physician visits, 5.c) Benign diseases, 5.d) Curiosity/interest, 5.e) “Testing” SCAs. The first subcategory 5.a) *Apprehension* covers all statements indicating that fear was the motivation to use SCAs. This includes, for example, the users’ anxiety that the symptoms or the disease could worsen. Another motivation using SCAs was to avoid physician’s visits. This motivation was summarised in the subcategory 5.b) *Avoid physician visits.* It covers visits considered unnecessary by the participants or when being abroad. Some used SCAs for everyday complaints or “trivialities”, summarised in 5.c) *Benign diseases*. One participant, for example, described the appeal of the SCA as being able to raise and reflect on trivial things that would otherwise not be discussed.

*“I mean, [the symptoms] are somehow trivial. I thought that was the attraction of the app, that the things that otherwise go by the board, […] that you then somehow reflect these trivial things more.” (NT 47)*

Others used it just out of curiosity, collected in the subcategory 5.d) *Curiosity/interest*.

*“I was just curious to see how advanced the technology is, that they could do something like that. Somehow it goes a bit in the direction of a “virtual doctor”. A bit like that. And I was simply interested: How good the algorithm is, so I downloaded it and tried.” (NT 42)*

As in this quote, curiosity often referred to the technology of the SCA, the algorithms behind it, or technical progress in general. In this context, thoughts were made about a virtual future. Other participants used the SCA to test it, collected in 5.e) *“Testing” SCAs*. This means that the users check whether the SCA works well or at all.

### Risks

The sixth category “Risks” summarises the concerns of the participants in the context of their SCA use and has four subcategories: 6.a) No concerns, 6.b) Subsequent assignment of responsibilities, 6.c) Concerns, and 6.d) Sense of security. The participants’ perceptions of potential risks associated with SCAs varied widely. While some participants indicated various risks, other participants had no concerns at all. The latter was summarised in the subcategory 6.a) *No concerns*. Here, the participants denied having concerns, for example regarding their (health) data. One argument raised by the participants was their own laziness. Data protection regulations, such as the GDPR, and the reputable impression of SCAs were described as further reasons not to worry. In addition, potential concerns about data protection were relativised by referring to other major problems of data misuse on the internet and the participants’ use of further apps.

*“For me, this data protection is a bit overdone […] I don’t care if Mr. Zuckerberg knows, for example, that I have asthma, because he can’t do anything with that. If it makes him happy, then he should know. I didn’t question that. Of course, I also read that … You can already see that in the installation. Well, I’m not someone who reads through ten pages of something here, that’s too exhausting for me.” (NT 39)*

In the case of incorrect SCA findings and recommendations the participants took different positions regarding the question of responsibility. A few participants saw the responsibility with the SCA.

*“It’s also the responsibility of the SCA. So, on the one hand, of course, there is the danger that it causes unnecessary panic in people, but on the other hand …. if the SCA says “heartburn is harmless” and people stay at home and the next day they are dead or have something serious because they just failed to go to the doctor in time. And that’s a difficult balancing act”. (NT 06)*

Although some users also considered the SCA to be responsible, they considered this attribution to be a difficult decision. On the one hand, the SCA could be too cautious and thus cause fear among users, but if it warns too little, this could have serious consequences for the user. Most participants saw the reason, and therefore responsibility, for incorrect SCA findings and recommendations in the incorrect input of the app users. The participants reasoned that the questions by the chatbot were not understood correctly by the users, that their data input was incorrect or dishonest, and that users exaggerated or understated their symptoms.

*“If a wrong input is made, it could of course be that someone, if he is not honest, won’t go to the doctor, but has something life-threatening, and then - let’s take the worst-case – pass away a day later, because the app suggested something harmless, and he actually has something serious, because he wasn’t honest. So, everyone has to be aware that if they don’t give honest information, they can’t expect a correct answer.” (NT 19)*

Although most participants saw the responsibility on the side of the SCA users, the participants had difficulties to position themselves. All statements in this direction were collected in the subcategory 6.b) *Subsequent assignment of responsibilities*.

The subcategory 6.c) *Concerns* collects all descriptions of the participants about risks and concerns in the context of their SCA usage. The participants described a wide variety of concerns. Missing aspects or questions during the data query, such as intake of medication, were named, which could then lead to incorrect findings and following incorrect user behaviour. Mental distress and symptom intensification through SCA usage were further concerns described by the participants. Examples of mental distress include nervousness, impatient, or anxiety during SCA use. One participant described, for example, that an already existing anxiety could intensified while using the SCA.

*“But if you now, for example, just now, with this rash don’t know what that could be and are already a bit scared, then you are already a bit nervous with the app and think so: Okay, okay, what could come out? What could it be?” (NT 41) *Some worried about a non-critical use by app users, and possibly following self-treatment, which would not be embedded in a medical or professional relation. In this context, it was questioned by some participants whether SCA users place too much trust in the app.

*“Maybe people trust the app too much. At the end of the day, it’s still an app and, yes, it doesn’t actually replace a doctor’s visit. I think that it might also be a risk that you rely on it too much.” (NT 41)*

The participants argued that the risk-averse recommendations of the SCA could lead to unnecessary consumption of limited healthcare resources (over-triage).

*“I think the app is very cautious. I have the feeling that the app sends you quickly to the doctor […] if there are now people who question the app’s results less, then the healthcare system, hospital, doctor, could be overloaded with things that are not necessarily worth treating or were not questioned. Because I simply saw for myself, yes, the app is a bit more cautious.” (NT 44)*

In this context, the SCA was frequently described as overly cautious or excessively hasty in its judgment. Such a tendency could present challenges for healthcare systems, particularly concerning resource constraints and system overload. These issues could be exacerbated if the SCA users don’t engage critically with its recommendations.

*“I just thought that this one reaction or the one response from the app was really exaggerated. And I think it’s questionable whether you send people like that to emergency rooms and use resources that we all know are very limited.” (NT 39)*

At the same time, the participants claimed the waiver of benefits as a potential risk for the SCA users (under-triage). Life-threatening situations were repeatedly mentioned as an example.

*“Situations can also arise where someone doesn’t go directly to the doctor, even though it really could be life-threatening and they urgently need medical help. So that is of course the reverse side of it.” (NT 39)*

As this quote shows exemplarily, participants were aware of both sides. Although the participants mentioned a lot of risks through SCA use, the participants felt in general safe using SCAs. All descriptions of the participants in this direction were summarised in the subcategory 6.d) *Sense of security*.

### SCA-use for others

The main category “SCA-use for others” was also directly derived out of the interview material and has three subcategories: 7.a) Addressee, 7.b) Problems, and 7.c) Unproblematic. The participants reported throughout the interviews that they used SCAs not only for themselves but also for others. Especially family members, such as partners and own children, but also friends were mentioned. The participants had entered symptoms from others in their SCA to get findings for them or used the SCA together. For example, one participant reported that since they had more than one child and in general children had very often complaints, they could check with the SCA whether physician visits were necessary or not (NT 21). A further group of addresses were technology-critical people. One participant described, for example, that she had used the SCA for someone who was sceptical about using smartphones and apps in general (NT 42). These different addresses were collected in the subcategory 7.a) *Addressees*. Some participants mentioned problems regarding privacy when entering personal data and symptoms of others into the app. For example, one participant described the use of the SCA as appropriate for a family member, but rather inappropriate for a person she did not know well, due to the perception of (health) data as private.

*“It was my mom, so it was very familiar. If I had done it, I think, for someone I didn’t know well, I think I would have been uncomfortable. And I think I would put the smartphone in his hand and say, ‘You type. I don’t need to see exactly what you’re typing in.’” (NT 42)*

In addition to concerns about privacy, participants raised questions of responsibility regarding SCA results and following recommendations for others.

*“I was very unsure when I made recommendations for others. So, I used the SCA for other people … because then that happened somehow on my recommendation. And I then somehow had the feeling, if a doctor’s visit is not recommended, and there is still something serious, then […] I have done something wrong.” (NT 42)*

Although recommendations for others were made by the users based on SCA findings, uncertainties were expressed in this regard. The uncertainties related primarily to whether it was acceptable to make a recommendation for others. Because, if the third party were to suffer harm as a result of the recommendation, then the responsibility would be localized to the person who had made the recommendation and this did not seem comfortable to the SCA users. The concerns of the participants regarding their SCA use for others were collected in the subcategory 7.b) *Problems*. While some participants assessed the SCA use for others as problematic, others saw this use as unproblematic. The latter statements were summarised in the subcategory 7.c) *Unproblematic*.

## Discussion

This qualitative study shows the broad scope of subjective experiences of SCA users. The study provides an overview of the various topics that users found relevant and addressed in relation to their SCA use. Thus, a wide spectrum of subjective experiences, including attitudes, expectations, motivations, and concerns is presented. The results demonstrate not only the broad spectrum of experiences but also inconsistent aspects. They show neither uniform expectations nor a consensus on the positive and negative effects of SCA. Instead, there are very different assessments of this new technology. Contradictory statements can be found in the categories, but sometimes even within one interview. Often there is not a concrete evaluation or specific argument, but a continuum of statements. These different and sometimes contradictory evaluations may be due, among other things, to the different individual situations of the users. This shows that it is very difficult to assess new technologies such as SCAs in general, but instead the individual, social, and cultural contexts must be taken into account.

More attention should also be paid to the structural level and the situatedness of these apps, as mobile health technologies may be discriminatory or worsen structural injustices [27]. Health apps can bring new challenges for health equity, as they may not be designed for everyone who could benefit from their use, for example, due to digital literacy or language. In addition, power imbalances, e.g. (implicit) racism and sexism, can influence the design of health apps. It is therefore important for a comprehensive ethical analysis to consider the specific social context and the structural-social processes in which such health apps are used. Although these structural dimensions were less discussed by the participants in our study, there is more and more discussion in the ethical literature on the systemic and structural problems in which digital health technologies are used [27–29]. This debate needs to be better linked to the specific requirements of SCAs, meaning for example, that the content and the functions of SCAs should be evaluated through the lens of structural dimensions such as structural or epistemic injustice.

Another important concern with AI-based systems, such as health apps, is algorithmic bias. There are many sources and different types of algorithmic bias [30]. A more specific understanding of bias in the sense of discrimination against certain persons or groups is widely recognized in the scientific literature on AI, including much of the health literature [31]. Interestingly, the participants did not discuss algorithmic bias, but bias by their user behaviour. Since the interviewed persons in our study did not mention algorithmic bias as a concern, it may suggest that the importance of this issue for researchers could not necessarily translate to groups in (clinical) practice. Another interpretation could be that the participants were not aware of this issue. Further research would be important to understand how bias in the context of AI and health is perceived and categorized by users of mobile health technologies. 

However, the contexts of the users and the range of their experiences have received little attention in the existing discussions. A recent review showed the absence of comparable empirical data in the debates on SCAs [6]. Further studies criticised the missing real-word data in these debates [e.g., 10; 12; 13]. These critiques indicate that the existing debates might not reflect the diverse users’ views on SCA. This study, in contrast, shows the plurality of the users’ experiences. This has implications for the ethical, social, and legal debates on SCAs. Also, it impacts empirical research: if absolute categories do not reflect the diversity of opinions and the same factor can both be regarded as negative or positive depending on the users’ context, questionnaire or interview guideline design needs to consider this. To gain a better understanding of the complexity of user experiences in digital innovations such as SCAs, context-related investigations are essential to identify the factors that led users to consider the usage of SCAs as beneficial, while others evaluated it more negatively.

The limited consideration of SCA user experiences in the existing discussions also opens up a debate about patient knowledge and epistemic injustices within digitized healthcare contexts. Epistemic injustice refers to situations where individuals or groups are harmed in their capacity as knowers [32]. Epistemic injustice can manifest in different ways. Common forms are downgrading certain persons’ testimonies and interpretations, their ability to contribute to knowledge, or not listening to them [32]. Patients’ testimonies and interpretations are often dismissed as irrelevant, too emotional, or time-consuming and often ignored, rejected, or subordinated to the authority of healthcare professionals [33, 34]. Regarding digitized healthcare, there are concerns about epistemic injustice, particularly when the knowledge of marginalized or vulnerable patient groups is dismissed by technology systems or in the development of these systems because digital technologies can reinforce existing epistemic inequalities and injustices in the health sector [35–37]. Whether SCAs promote or prevent epistemic injustices is another very interesting question. The point here, however, is that the experiences of users and patients should be given more attention in debates about SCAs. As the digitization of healthcare continues to advance, more ethical and social research must be developed to address these issues and ensure that patient knowledge is included in the debates and the development of digitized healthcare. 

The users’ subjective experiences are one step in assessing whether the promises of SCAs contributing to better healthcare (systems) are being fulfilled or not. Since the views in our interviews diverge widely, it is difficult to follow that the expectations of the users are met (or not) by the SCA. Again, it depends strongly on the individual users’ prior experience, knowledge, and situation. In addition, satisfaction with a health tool does not necessarily mean that this tool is helpful or beneficial, but rather that this tool might still or continue to be used. Satisfaction can thus be seen as a prerequisite for realising potential benefits of SCAs. Whether SCAs are helpful or beneficial in the medical or clinical context, however, is another question. Although SCAs promise to advance diagnostic practices, diminish misdiagnosis, and guide patients through healthcare systems more effectively, there is currently little empirical evidence to support this. Nevertheless, empirical evidence on expectations and satisfaction is indispensable for ethical evaluation, because low satisfaction can lead to the application not being used, whereas high satisfaction can lead to the application being overused. Further qualitative studies are required to get a better understanding of the underlying behavioural and motivational processes outlined in this study. At the same time, large-scale quantitative studies would be necessary to go further regarding representation and more breadth. However, to determine whether SCAs can benefit patients and healthcare systems, it is primarily necessary to determine the diagnosis and triage accuracy of SCAs, the effects of SCAs on patients’ care-seeking behaviour and the safety and appropriateness of those decisions. Initial empirical studies [e.g., 7; 8; 10; 38] and guidelines for evaluating symptom checkers already exist [e.g., 39], but need to be further developed.

The study reveals that the users’ own judgement, as well as the physicians’ opinion, are placed over the app findings by the users. This result is contrary to the implicit but often underlying question in debates about whether SCAs could replace medical professionals or even lead to a devaluation of the professions [e.g., 38; 40; 41; 42]. This implicit concern in the literature is not confirmed in our qualitative study. This again highlights that the debates on SCAs have so far been conducted without the views of the users. Further ethical, social, and legal debates on SCA should therefore incorporate the users’ perspective to a greater extent. Further qualitative research should go even more into detail to allow for more nuanced debates. For example, if the concern of replacement is stated in the literature, it needs to be examined more precisely which user groups are referred to. More empirical research would be helpful to understand whether users’ characteristics have an impact on SCA use. Specifically, whether the characteristics influence how the users rank the app findings compared to their own evaluation or that of a physician. In addition, since the SCA users decide whether or not to accept the app’s findings, (e)health literacy seems to be a crucial factor in these decisions. Further research should investigate the role of (e)health literacy in these decision-making processes. In a study by Miller et al. [12] most SCA users responded that using SCAs would not change their decisions about what to do next (e.g., go to a GP). Another study by Fraser et al. [10] discussed the potential that patients would change the urgency level of care they sought based on SCA findings. It would be interesting to investigate whether the confidence to put their own opinion above the SCA findings is related to user characteristics, such as (e)health literacy, and to what extent an ongoing use of the app would influence this self-confidence.

The category “App use for others” evolved in the analysis process. App use for third parties or specific social contexts (friends, spouse, family) are not discussed in the literature on SCA. Even in the broader debates around health apps, most considerations focus on the individual app user and relational aspects are seldom considered [43]. The examples of app use for others in our interview study therefore raise hitherto unexplored ethical aspects. Unsolicited SCA use for third parties, for example for one’s own children or partner, can be further discussed from a paternalistic point of view. In addition, ethical questions regarding autonomy and privacy come up because sensitive personal information, such as health data, are needed for the SCA input. In the interviews, the participants also described situations in which the individuals used the SCA together because they preferred to talk about their symptoms to their friend or partner rather than to a physician, for example, out of shame. In these cases, normative questions arise about privacy, intimacy, the physician’s role and trustworthiness. A different example is when the SCA user replaces the physician, i.e., makes a recommendation for a third person via the SCA. This case also raises questions about the physician’s role, medical authority, and responsibility. Health apps such as SCAs can provide inaccurate or incomplete information, which may lead to false recommendations. Who is responsible if a friend or a parent uses the SCA making a recommendation that leads to harmful behaviour of a third individual? The examples from the interviews show that SCA use for others is a complex theme, which raises a lot of ethical questions.

### Strengths and limitations

The qualitative and explorative study design affords a broad understanding of the subjective experiences of SCA users. The strength of the analysis lies in its wide scope and detail. The qualitative semi-structured interviews and the conduct of the interviews in tandem allowed the breadth. Ongoing discussions of the analysis in the research group with different disciplinary backgrounds helped to investigate the study subjects from different perspectives. In addition, a member check was used to check the relevance of the results for the people concerned. Internal methods-workshops in the CHECK.APP project and an external advisory board served as further tools to validate the results of the interview analysis.

Various recruitment tools were used in the overall project to obtain a representative sample. In the first phase of the project, however, only German citizens were contacted. Although further recruitment was carried out (e.g., via social media), only German-speaking people were included in the sample. It would be interesting to compare interviews with participants from other cultural contexts with those of the present study. Although different recruiting tools were used to ensure the inclusion of participants of differing gender, in different parts of their lifespans, and with varying experiences, most participants were between 20 and 29 years and well-educated. The results of our study should be seen against the background that young well-educated (seemingly cisgender) users made up a large part of the study sample. For example, the participants’ positive evaluation of their skills and the preference of their assessment in comparison with the SCA findings might be different for a more diverse sample. As there is currently too little empirical data about SCA user characteristics, it is difficult to say whether our sample is representative of typical SCA users [44]. Further studies should investigate the extent to which social categories such as age, gender, ethnicity, and education of users are related to the use of SCAs.

The sample included only participants who have already experienced the symptom checker Ada. The participants might have reported other aspects and experiences when talking also about other symptom checker apps. Ada was used in the study as an example for a SCA, as it is one of the most popular SCA in Germany. It may be that some study participants rated the app more positively since it is being researched within an independent academic institution that they trust. At the same time, it is possible that some participants were reluctant to make critical comments about the Ada app because our independence seems not always clear to them. Although we repeatedly indicated (in written and oral form) that we were conducting research independently of Ada, this may not always have been present to the study participants. In addition, the health status of the participants may have had an impact on the interview results. Depending on whether the participants were in a phase of illness or not, they might evaluate the SCA differently. Moreover, the pandemic situation also might have had an influence on the study results. For example, it was not possible to conduct the interviews in person. Instead, we conducted the interviews via video call. The nuances of body language and other nonverbal cues associated with face-to-face interaction may be lost over video calls, and trust may be more difficult to establish. In addition, the tandem design of the interviewer may have influenced the interviewed persons. It could be that the interviewed person felt less comfortable talking about their experiences because there were two researchers present.

Finally, there were some difficulties in the analysis and interpretation of the data. The clear definition of the search units, for example “motivations” and “expectations”, emerged as a major difficulty within the current study. There are no hard criteria within research literature regarding the formulation of what are motivations and what are expectations, and how these aspects can clearly be distinguished from each other. In order to resolve this situation and to obtain the most comprehensive picture possible, we used broad definitions and allowed multiple coding in the coding process. The chosen method allows the coexistence of attitudes, expectations, motivations, and concerns. Further empirical research would be important to better understand these distinctions in the context of health app use and how these categories are connected with each other.

## Conclusion

The present qualitative study investigates the subjective perspectives of SCA users, in particular, their expectations and assessments of SCA usage. The aspects identified in the analysis demonstrate the immense scope of different experiences and evaluations. In addition, it shows the users’ uncertainty regarding the advantages and disadvantages of SCA use and corresponding ethical implications. The interviews also emphasise an ethical dimension of SCAs which has scarcely been discussed in the literature: the use for third parties. The interviews show that SCA use for others is a complex theme, which raises various ethical aspects such as autonomy and paternalism, intimacy and privacy, as well as trustworthiness. For example, the triangle between the recommendation of the SCA, the SCA owner, and a third person raises questions of responsibility. Furthermore, the physicians’ role in this constellation is not clear. These normative relational issues seem to be underexposed in the literature on health apps yet, particular on SCA. Relational aspects should be paid more attention in the ethical debates on mHealth, but also in the development and distribution processes of health apps such as SCA. It is important for app developers, providers, and regulators to be aware of these relational ethical issues and take steps to address them in order to ensure that health apps are developed and used in a responsible way that protects the rights of the individual user, but also of third parties involved.

### Electronic supplementary material

Below is the link to the electronic supplementary material.

Supplementary Material 1: CHECK.APP User Interview Guide (English)

## Data Availability

The interview material generated and analysed during the current study are not publicly available due to privacy issues. The first author can be contacted for access to the raw data analysed in the study. Contact details: regina.mueller@uni-bremen.de.

## Abbreviations

GP: General Practitioner
SCA: Symptom Checker App
