# Supplementary material for: “That’s just Future Medicine” - a qualitative study on users’ experiences of symptom checker apps
Source: BMC Med Ethics. 2024 Feb 16;25:17. doi: 10.1186/s12910-024-01011-5 (PMC10874001; doi:10.1186/s12910-024-01011-5)
Supplement: Supplementary file 1 — Supplementary Material 1: CHECK.APP User Interview Guide (English) [file 12910_2024_1011_MOESM1_ESM.docx]

**Supplement 1: CHECK.APP User Interview Guide (English)**

| Interview part | Interviewer | Topics/Themes | Other interviewers/listeners |
| --- | --- | --- | --- |
| Introduction | Together | Welcome, role distribution, information about the procedure and structure of the interview  Short self-introduction of the interviewer and the interview partner  Information about independence from App manufacturer |  |
| Consent | Together | Information about video recording, data storage on UKT computers, data security, transcription, and deletion of the video files  Ask for consent to switch on the recording |  |
| Narrative part | IAIV | **How did you come to participate in the study?**  **What was it like in the time before the diary study, what experiences did you have with the app until then?**   - - e.g., first experience with the app   **Think about the 6 weeks during which you had filled in the usage diary. What do you remember specifically from that time?**   - - What kind of day was it?   - How did it go on?   - How did you feel?   - Who else was there?   **In general, how did you feel during the six weeks?**  **What happened after you sent the diary back to us?** | Notes interesting questions/topics for the normative part |
| Change-Over | Together | Questions/topics noted by the other interviewer (if these do not come later) |  |
| Normative | Ethics/law | **What expectations did you have before using the app?**   - Were these expectations met or disappointed? Why?   **Pros and cons of using the app?**  **Think back to chatting with the app:**   - How was it for you to communicate with the app? - Some people communicate very openly with the app, while others tend to withhold information, repeat entries, and play with it. How was that for you? - **What differences did you notice in comparison to conversations with physicians/doctors? How do you evaluate these differences?**   **How did you perceive the results of the app?**   - Did you perceive the results as a "diagnosis"? - When you think about the app's recommendations, have you followed the app's recommendations? Why? Why not? - Did you perceive any risks in the context of the app? (e.g., concerning the suggested diagnoses or recommended actions?)   **What role does the app play for you in connection with visits to the doctor?**  Feel free to describe it using a specific situation that you have experienced.   - At what point did you decide to go to the doctor? - What influence does (did) the app have on your decision? - What influence does the information you get from the app have when you consult your doctor? (Do you mention the app?)   **How well do you feel taken care of by the app?**   - What does "feeling in good hands" mean for you in the context of using the app? - Did you have any concerns/ to what extent did you have concerns (e.g., providing personal health data, receiving wrong results or incorrect recommendations)? - Where do you see risks? | Statements/points related to episodic knowledge |
| Clarifying Questions | Ethics/Law | Further questions/points and terms to be clarified |  |
|  | IAIV | Further questions related to episodic knowledge |  |
| Ending | Together | **Have we forgotten something?**  **Is there anything else you would like to tell us?**  **Thank you very much for your participation!** |  |

**Bold: Main Questions /** *Italics: Follow-up Questions*
